# *Abelmoschus manihot* for Diabetic Nephropathy: A Systematic Review and Meta-Analysis

**DOI:** 10.1155/2019/9679234

**Published:** 2019-04-18

**Authors:** Liwei Shi, Ling Feng, Meizhen Zhang, Xiaowen Li, Yanan Yang, Yueying Zhang, Qing Ni

**Affiliations:** ^1^Department of Endocrinology, Guang'an Men Hospital, China Academy of Chinese Medical Sciences, Beijing 100053, China; ^2^Department of Health Care, Guang'an Men Hospital, China Academy of Chinese Medical Sciences, Beijing 100053, China; ^3^Beijing University of Chinese Medicine, Beijing 100029, China

## Abstract

Diabetic nephropathy (DN) is the leading cause of end-stage renal disease (ESRD). Many trials have shown that* Abelmoschus manihot* could further improve proteinuria and protect kidney function in patients with DN when added to a renin-angiotensin system (RAS) blocker. A systematic assessment of the efficacy and safety of* A. manihot* in DN is essential. Eight electronic databases were searched to identify eligible trials published from inception to December 2017. The Cochrane Risk of Bias Tool was used to evaluate the methodological quality of eligible studies. Seventy-two studies with 5,895 participants were identified. The methodological quality of included studies was generally low. The results indicated that, compared to a RAS blocker, combined treatment of* A. manihot* with a RAS blocker was more effective for 24h urinary protein (24h UP) (mean difference [MD], -0.39 [95% confidence interval [CI], -0.46 to -0.33] g/d; P<0.00001), urinary albumin excretion rate (UAER)(MD, -19.90 [95% CI, -22.62 to -17.18] *μ*g/min; P<0.00001), 24h UP reduction rate (risk ratio [RR], 1.43; 95% CI, 1.26-1.63; P<0.00001), normalization of UAER (RR, 1.48; 95% CI, 1.29-1.70; P<0.00001), and serum creatinine (SCr) (MD, -7.35 [95% CI, -9.95 to -4.76] umol/L; P<0.00001). None of these trials reported the ESRD rate. No statistically significant difference occurred between* A. manihot* combined with a RAS blocker and a RAS blocker alone in estimated glomerular filtration rate (eGFR) (MD, 4.43 [95% CI, -1.68 to 10.54] mL/min; P=0.16).* A. manihot* did not increase the rates of adverse drug events.* A. manihot* in addition to a RAS blocker was effective and safe to further improve proteinuria and protect kidney function in patients with DN. However, due to the generally low methodological quality, significant heterogeneity, and publication bias, high-quality randomized controlled trials are required to confirm these findings before the routine use of* A. manihot* can be recommended.

## 1. Introduction

Approximately 20% to 40% of patients with diabetes mellitus (DM) will develop diabetic nephropathy (DN) [1]. Type 2 diabetes mellitus (T2DM) is the most common cause of chronic kidney disease (CKD) and end-stage renal disease (ESRD) in the developed world and is the second leading cause of ESRD after primary glomerular disease in China [2–4]. DM and CKD are independent risk factors of all-cause mortality as well as cardiovascular death [5, 6]. Diabetic kidney disease (DKD) poses the highest risk for death compared to DM or CKD alone [5, 6].

Management of DN requires a multifaceted approach, including a combination of lifestyle modifications and pharmacologic intervention. The effectiveness of current interventions remains limited given the number of patients who continue to have progression of their renal dysfunction, despite blood pressure and glycemic control, and the use of existing renin-angiotensin system (RAS) blockers. Retrospective analyses of clinical studies concerning DN demonstrate a strong relationship between the magnitude of albuminuria reduction and slowing of CKD progression as well as reduced cardiovascular event rates [7–12]. An angiotensin-converting enzyme inhibitor (ACEI) combined with an angiotensin receptor blocker (ARB) is not recommended due to the high risk of hyperkalemia and/ or acute kidney injury as well as no benefit in altering the natural history of DN [1, 10]. Recently, mineralocorticoid receptor antagonist (MRA) in addition to an ACEI/ARB treatment has been studied as a novel approach to further prevent the progression of DN. A meta-analysis by Mavrakanas et al. [13] reported that combined treatment with an ACEI/ARB and an MRA was effective in decreasing albuminuria compared to standard treatment with an ACEI/ARB in DN but increased the risk of hyperkalemia. Therefore, there is an urgent need for a new pharmacologic agent that could be effective and safe to further improve proteinuria and prevent the progression of DN.

Traditional Chinese medicine (TCM) has shown promising effects on the control of proteinuria, protection of renal function, and improvements in patients' clinical symptoms [14].* Abelmoschus manihot* has been in use for CKD in China for hundreds of years. Huangkui capsule, a single medicament of TCM extracted from the dry corolla of* A. manihot*, has been approved by China's State Food and Drug Administration (SFDA) for the treatment of chronic nephritis since 1999.* A. manihot* can ameliorate proteinuria and protect kidney function in patients with CKD, such as DN, immunoglobulin A nephropathy (IgAN), and membranous nephropathy, and is currently considered an important adjuvant therapy for CKD [15–20]. The major biologically active constituents are total flavones of* A. manihot* (TFA) [21]. Mechanistic studies applying* A. manihot* to the treatment of CKD suggest that the major effects are associated with improved immunological reaction, inflammation, renal fibrosis, and renal tubular epithelial injury [14, 22]. The results of previous meta-analyses preliminarily suggest that* A. manihot* could improve proteinuria and protect kidney function in patients with DN [16–19]. However, the evidence was very limited on the effect of* A. manihot* for DN due to a limited number of trials included, with poor methodological quality. A lot of novel data evaluating* A. manihot *in DN have been recently published. Therefore, we systematically analyzed the evidence on* A. manihot* in addition to a RAS blocker therapy in DN, focusing on its effect in albuminuria.

## 2. Methods

### 2.1. Data Sources and Searches

This systematic review was reported in accordance with Preferred Reporting Items for Systematic Reviews and Meta-Analyses (PRISMA) [23]. See File S1 in the Supplementary Material for the PRISMA 2009 checklist for this article. The review protocol was registered with the International Prospective Register of Systematic Reviews (PROSPERO registration no. CRD42018087182, available at http://www.crd.york.ac.uk/PROSPERO/display_record.php?ID=CRD42018087182). The Cochrane Central Register of Controlled Trials (CENTRAL) on the Cochrane Library, PubMed, EMBASE, Chinese National Knowledge Infrastructure database (CNKI), Chinese Biomedical Literature database (CBM), Chinese Scientific Journal database (VIP), and Wan Fang database were searched to identify eligible trials published from inception to December 15, 2017. Ongoing registered clinical trials were searched at ClinicalTrials.gov (https://www.clinicaltrials.gov). The articles were not restricted based on language. All included studies were subjected to the same quality assessment.

The search terms were as follows: Flos Abelmoschus manihot, Abelmoschus manihot, Abelmoschus moschatus Medicus, Abelmoschus, okra, Huangkui, Huangkui capsule, huangshukui, diabetic nephropathy, diabetes mellitus, diabetic, kidney disease, renal disease, diabetic kidney disease, diabetic renal disease, albuminuria, randomized controlled trial, controlled clinical trial, randomized, randomly, and trial. See File S2 in the Supplementary Material for an example of the full electronic search strategy. Two authors (L. W. Shi and M. Z. Zhang) performed independently the literature search. Disagreements were resolved by discussing with a third party (Q. Ni and L. Feng).

### 2.2. Study Selection

Eligible trials were listed and assessed independently by two reviewers (L. W. Shi and M. Z. Zhang) using predefined inclusion criteria. Studies were included if they met the following criteria: (1) it was randomized controlled design; (2) patients were with type 1 or type 2 DM and DN (defined as at least 30 mg of albuminuria in a 24h urine collection or urinary albumin excretion rate (UAER) of at least 20 *μ*g/min); (3) participants should have received an ACEI or an ARB throughout the study as standard treatment. To evaluate the effect of concomitant* A. manihot*, a subset of patients in each study should also receive* A. manihot* in addition to standard RAS blockade; (4) the primary outcome measures included 24-h urinary protein (24h UP), ESRD rate and estimated glomerular filtration rate (eGFR). The secondary outcome measures were UAER, improvements in 24h UP reduction rate (defined as the proportion of 24h UP decrease in protein excretion ≥50% of the baseline at the end of the study), normalization of UAER (defined as the proportion of UAER <20 *μ*g/min upon study completion), serum creatinine (SCr) and adverse drug events (ADEs); (5) the studies included available and relevant data; and (6) the studies were not restricted based on publication language.

Excluded from the meta-analysis were duplicated publications, studies with unavailable or incorrect data, and articles not reporting outcomes of interest. Also excluded were studies enrolling fewer than 10 participants, quasi-randomized controlled trials (e.g., allocation using alternation, the sequence of admission, case record numbers), and nonrandomized controlled clinical trials. Studies using combination RAS blockers as background therapy or* A. manihot* coupled with any other TCM drugs were excluded to avoid confounding information.

### 2.3. Data Extraction and Quality Assessment

Two authors (X. W. Li and Y. N. Yang) independently extracted information on the patients as well as on the methods, interventions, outcomes, and results using a predesigned data extraction form. The data extraction form included the following items: name of first author, year of publication, total number and number in both groups, gender and mean age, baseline characteristics, method of randomization, allocation concealment, incomplete outcome data, selective reporting, blinding, interventions, and outcomes.

The methodological quality of randomized controlled trials (RCTs) was independently assessed by two authors (M. Z. Zhang and Y. Y. Zhang) via the Cochrane Risk of Bias Tool [24]. Each study was respectively categorized as “low risk of bias”, “high risk of bias”, or “unclear risk of bias”. Authors were contacted by e-mail to obtain further data and verify the methodological quality when necessary. The Grading of Recommendations Assessment, Development, and Evaluation (GRADE) methodology was used to assess the quality of the evidence of each outcome. Any disagreement was settled by mutual discussion with a third author (Q. Ni and L. Feng).

### 2.4. Data Synthesis and Analysis

Dichotomous outcomes were pooled using risk ratio (RR) and 95% confidence intervals (CIs) and continuous outcomes were pooled using mean difference (MD, defined as the difference between study groups at the end of study) and 95% CIs. A random-effects model was used to pool the data. Statistical heterogeneity was assessed with the I-square (I^2^) statistic [25]. The I^2^ statistic of ≤50% referred to low statistical heterogeneity, while >50% was considered as substantial statistical heterogeneity. Publication bias was performed and evaluated using funnel plots, if the group included >10 trials [26]. Sensitivity analysis was assessed by excluding lower quality trials and repeating the meta-analyses to examine the effects of these study subgroups. We had no prespecified plan of subgroup analysis. Meta-analysis was performed by using Review Manager Version 5.3. All tests were 2-tailed, and P<0.05 was considered statistically significant.

## 3. Results

### 3.1. Included Studies and Trial Characteristics

**Figure 1:**
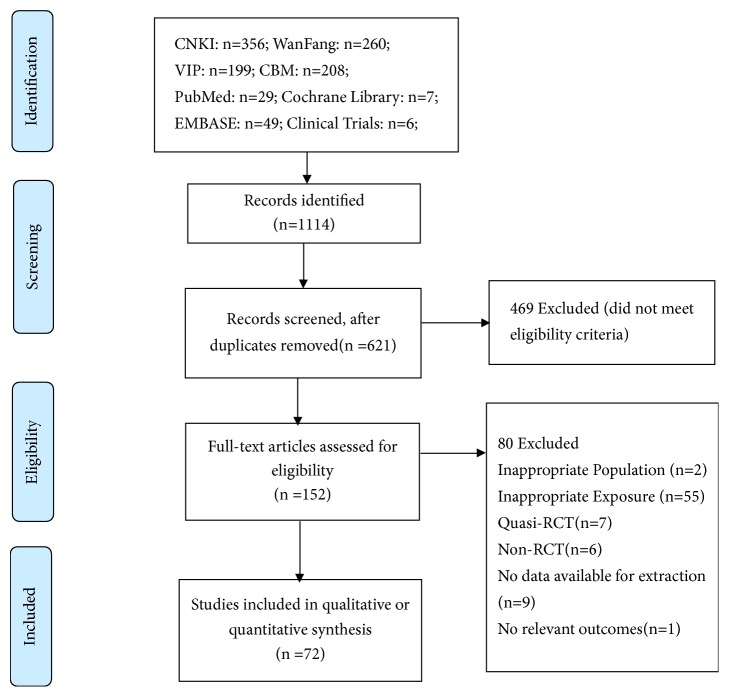
Flow diagram of study selection.

A flow diagram of study selection is shown in Figure 1. During the initial electronic search, 1,114 articles were identified, of which 962 were excluded including duplicates and irrelevant studies. The full texts of the selected 152 trials were retrieved, and after detailed evaluation, 72 RCTs [27–98] were finally selected for meta-analysis; of these, 28 met the inclusion criteria from 4 previous meta-analyses [16–19]. Authors were contacted by e-mail for additional outcome data; however, no reply was received.

The baseline characteristics of DN patients are presented in Table 1. The 72 studies included a total of 5,895 patients followed-up from 4 to 24 weeks. The treatment and control groups consisted of 3,000 and 2,895 patients, respectively. Sample size of the included trials ranged from 40 to 200. The mean age reported for participants in these studies ranged from 36 to 69 years, and the proportion of males ranged from 33.3% to 69.2%. The average baseline protein level in urine was 1.94 g/d (0.14-6.2 g/d). The median follow-up for 24h UP was 12 weeks.* A. manihot* in the form of a Huangkui capsule (Jiangsu SuZhong Pharmaceutical Group Co., Ltd.) was given orally at 3.0 g 3 times daily in one trial [27], orally at 2.5 g 3 times daily in 67 trials [28–41, 43–47, 49–64, 66–85, 87–98], and orally at 2.0 g 3 times daily in 3 trials [42, 48, 65]. In one study [86], Abelmoschus alcohol extract was given orally at 0.4 g, 3 times daily. A range of RAS blockers were used: in 13 (18.06%) studies [27, 28, 36, 44, 48, 49, 68, 69, 71, 73, 75, 86, 96] ACEI (Captopril, Enalapril Maleate, Fosinopril, and Benazepril) was used; in 52 (72.22%) studies [29–31, 33–35, 37–42, 45–47, 50, 51, 53–59, 61, 62, 64–67, 70, 76–85, 87–95, 97, 98] ARB (Valsartan, Telmisartan, Candesartan, Irbesartan, and Losartan) was used; and in 7 (9.72%) studies [32, 43, 52, 60, 63, 72, 74] either ACEI or ARB was used. All other concomitant therapies were comparable between study groups. Trials were all single-centered studies published from 1995 to 2017 and were conducted in China and published in Chinese.

### 3.2. Risk of Bias Assessment

**Figure 2:**
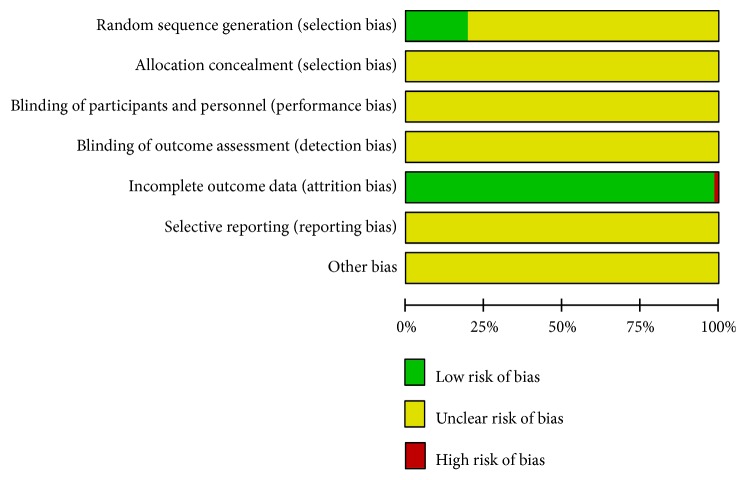
Risk of bias graph.

**Figure 3:**
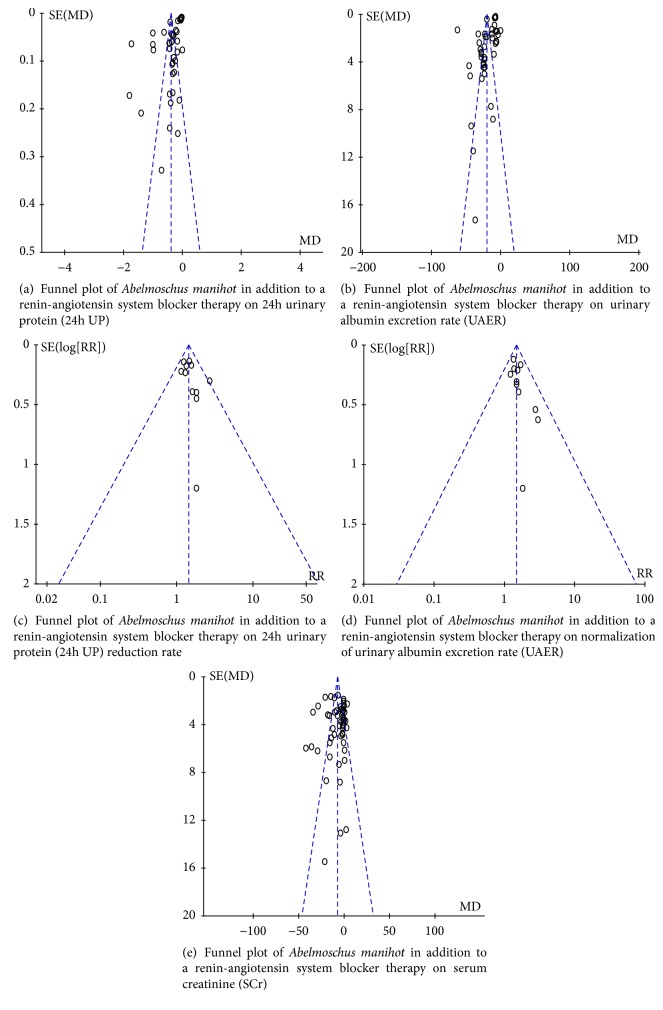
Funnel plots.

A summary of study quality is presented in Figure 2. The methodological quality was generally poor. All trials were reported to be randomized, but only 14 (19.44%) trials [28, 30, 35, 45, 47, 48, 52, 61, 65, 68, 75, 82, 88, 94] described adequate sequence generation. None of the included trials mentioned the methods for allocation concealment, the blinding of participants and personnel, and blinding of outcome assessment. Risk of attrition bias (incomplete outcome data) was detected in one [87] of all included trials, with a high risk status. Selective reporting and other potential sources of bias were unclear. Sensitivity analysis was not performed since all included trials were generally of low methodological quality. The funnel plots based on 24h UP, UAER, and SCr were asymmetrical, showing that publication bias might affect the results of this meta-analysis. The funnel plots constructed for improvements in 24h UP reduction rate and normalization of UAER were both nearly symmetrical, showing that publication bias might not affect the results of this meta-analysis. Funnel plots based on the primary and secondary outcomes are respectively elaborated in Figures 3(a), 3(b), 3(c), 3(d), and 3(e).

### 3.3. Effects of Interventions

#### 3.3.1. 24-h Urinary Protein (24h UP)

**Figure 4:**
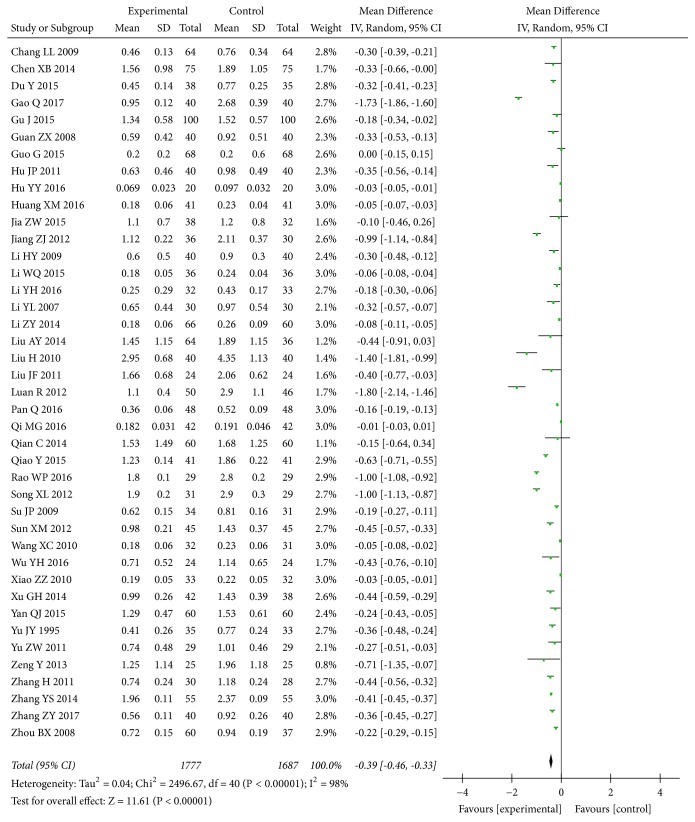
Effect of* Abelmoschus manihot* in addition to a renin-angiotensin system blocker therapy on 24h urinary protein (24h UP).

Data regarding the effect of combined* A. manihot* with a RAS blocker compared to a RAS blocker on 24h UP were available from 41 [27, 28, 32, 34–37, 39–44, 46, 48, 49, 52, 56–58, 60, 63–65, 69, 71, 73–76, 78–80, 84, 86–89, 92, 93, 96] of 72 trials, including 3,464 participants. The meta-analysis indicated that* A. manihot* plus a RAS blocker was associated with significant reductions in 24h UP level compared with a RAS blocker alone at the end of the study (MD, -0.39 [95% CI, -0.46 to -0.33] g/d; P<0.00001; Figure 4). There was evidence of significant heterogeneity across these trials (I^2^ =98%; P for heterogeneity <0.00001; Figure 4).

#### 3.3.2. End-Stage Renal Disease (ESRD) and Estimated Glomerular Filtration Rate (eGFR)

**Figure 5:**
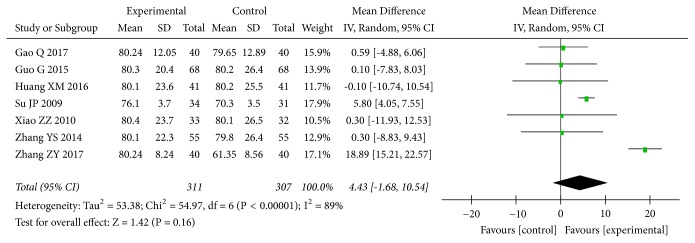
Effect of* Abelmoschus manihot* in addition to a renin-angiotensin system blocker therapy on estimated glomerular filtration rate (eGFR).

None of the included trials assessed the ESRD rate. Seven trials [34, 37, 41, 74, 79, 92, 93] with 618 patients assessed the effect of* A. manihot* plus a RAS blocker on eGFR in patients with DN. The results indicated that there were no statistically significant differences between* A. manihot* plus a RAS blocker and a RAS blocker alone in eGFR (MD, 4.43 [95% CI, -1.68 to 10.54] mL/min; P=0.16; Figure 5). There was evidence of significant heterogeneity across these trials (I^2^=89%; P for heterogeneity <0.00001; Figure 5).

#### 3.3.3. Urinary Albumin Excretion Rate (UAER)

**Figure 6:**
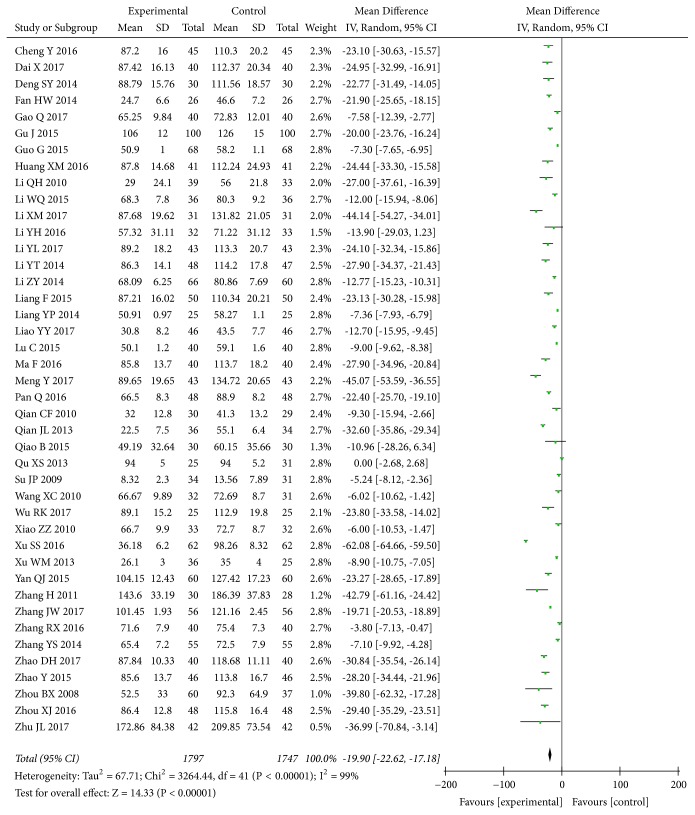
Effect of* Abelmoschus manihot* in addition to a renin-angiotensin system blocker therapy on urinary albumin excretion rate (UAER).

The effect of* A. manihot* on UAER level was reported in 42 trials [29–31, 33–35, 37, 41, 45–48, 50–55, 59, 61–63, 66–68, 70, 74, 76, 77, 79, 82–84, 89–92, 94–98], including 3,544 participants. The meta-analysis indicated that, compared to a RAS blocker alone,* A. manihot* combined with a RAS blocker was associated with a greater decrease in UAER (MD, -19.90 [95% CI, -22.62 to -17.18] *μ*g/min; P<0.00001; Figure 6). Again, there was evidence of significant heterogeneity across these trials (I^2^=99%; P for heterogeneity <0.00001; Figure 6). In addition, two [45, 91] of 42 trials reported that trial duration per patient was 20 weeks, with 8 weeks of treatment and 12 weeks of follow-up without treatment. The MD of UAER between study groups at the end of follow-up was assessed again and still less in the treatment versus control groups (one trial [45]: MD, -33.00 [95% CI, -42.93 to -23.07] *μ*g/min; p<0.00001, and another one [91]: MD, -11.40 [95% CI, -14.91 to -7.89] *μ*g/min; p<0.00001), indicating that the effect of* A. manihot* on UAER might persist for 12 weeks after treatment.

#### 3.3.4. Improvements in 24h UP Reduction Rate and Normalization of UAER

**Figure 7:**
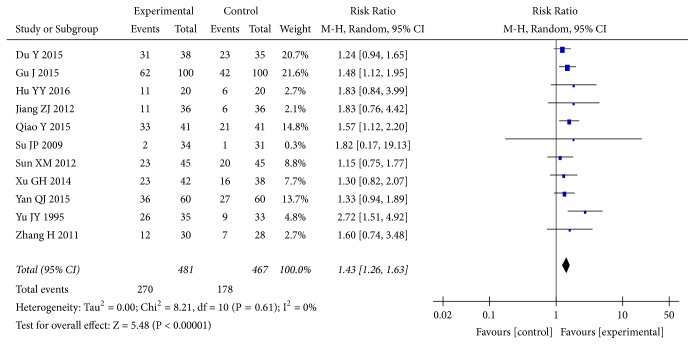
Effect of* Abelmoschus manihot* in addition to a renin-angiotensin system blocker in improving 24h urinary protein (24h UP) reduction rate. Improvements in 24h UP reduction rate, defined as the proportion of 24h UP decrease in protein excretion ≥50% of the baseline, at the end of the study.

**Figure 8:**
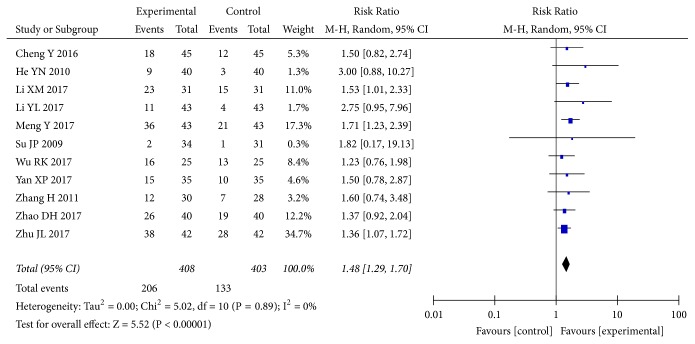
Effect of* Abelmoschus manihot* in addition to a renin-angiotensin system blocker in improving normalization of urinary albumin excretion rate (UAER). Normalization of UAER, defined as the proportion of UAER <20 *μ*g/min upon study completion.

Eleven [32, 35, 40, 43, 69, 74, 75, 80, 84, 86, 89] of the included studies reported changes in 24h UP reduction rate. The pooled results showed that* A. manihot* combined with a RAS blocker therapy was associated with significant improvements in 24h UP reduction rate compared with a RAS blocker alone (RR, 1.43; 95% CI, 1.26-1.63; P<0.00001; Figure 7). The normalization of UAER was reported in 11 trials [29, 38, 47, 50, 62, 74, 77, 85, 89, 94, 98] of 72 RCTs. The results showed that combined treatment of* A. manihot* and a RAS blocker was more effective in normalization of UAER (RR, 1.48; 95% CI, 1.29-1.70; P<0.00001; Figure 8) than a RAS blocker alone. Statistical heterogeneity was low for these outcomes, suggesting a consistent effect size across studies (I^2^=0%; Figures 7 and 8).

#### 3.3.5. Serum Creatinine (SCr)

**Figure 9:**
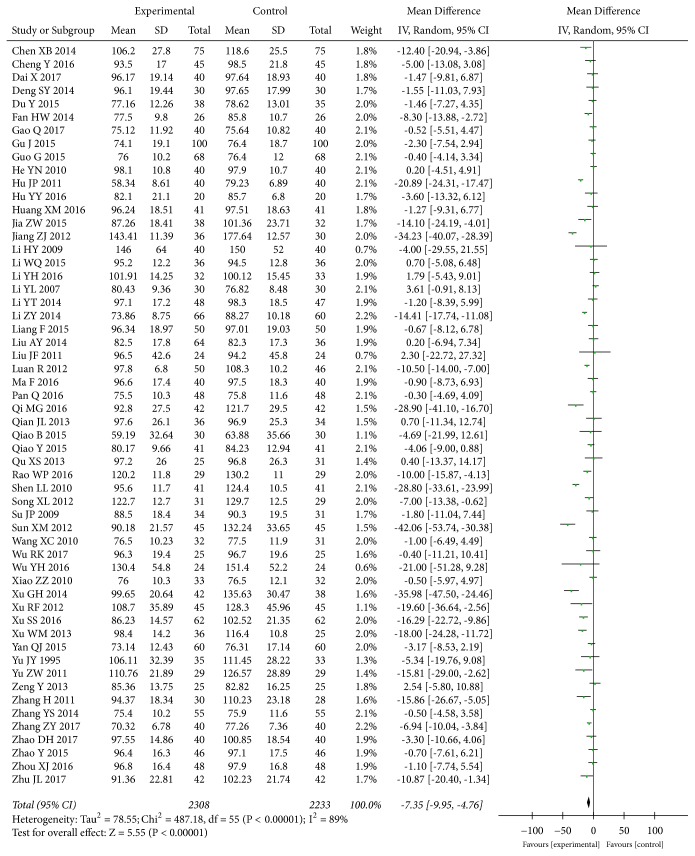
Effect of* Abelmoschus manihot* in addition to a renin-angiotensin system blocker therapy on serum creatinine (SCr).

Data for the effect of* A. manihot* combined with a RAS blocker compared to a RAS blocker on SCr level were available from 56 trials [28–35, 37–44, 46, 48, 49, 51–53, 56, 58, 60, 61, 63, 64, 67–84, 86–89, 92–95, 97, 98] including 4,541 participants. The meta-analysis indicated that compared with a RAS blocker alone,* A. manihot* combined with a RAS blocker led to a greater decrease in SCr level (MD, -7.35 [95% CI, -9.95 to -4.76] *μ*mol/L; P<0.00001, Figure 9), indicating that* A. manihot* was associated with improved kidney function. The I^2^ statistic based on the data for SCr exhibited significant heterogeneity (I^2^=89%, P<0.00001, Figure 9).

#### 3.3.6. Adverse Drug Events (ADEs)

ADEs were observed in 53 [28–32, 34–40, 42, 44, 45, 47–51, 53–56, 59–63, 67, 68, 70–75, 77, 78, 80, 83, 85–87, 90–98] of 72 RCTs; 27 [28, 34, 36–40, 47–50, 55, 56, 62, 63, 68, 71, 73, 74, 77, 85, 86, 92–94, 96, 98] of which reported that no ADEs occurred; 26 [29–32, 35, 42, 44, 45, 51, 53, 54, 59–61, 67, 70, 72, 75, 78, 80, 83, 87, 90, 91, 95, 97] reported that ADEs occurred, including gastrointestinal discomfort, dry mouth, headache, dizziness, liver injury, hypoglycemia, hyperkalemia, coughing, and hypotension. There were no statistically significant differences between study groups in all rates of ADEs except with headache, which was reported in 10 trials [29, 35, 51, 53, 61, 75, 80, 90, 95, 97] and occurred more commonly in the control group (RR, 0.29; 95% CI, 0.11-0.76; P=0.01; I^2^=0%). Twenty-one trials [29–32, 35, 44, 45, 51, 53, 59–61, 67, 70, 72, 78, 83, 90, 91, 95, 97] were included in the pooled RR for gastrointestinal discomfort (RR, 1.24; 95% CI, 0.72-2.13; P=0.45; I^2^=0%). Eleven trials [29, 31, 35, 51, 53, 54, 59, 61, 90, 95, 97] were included in the pooled RR for dry mouth (RR, 0.51; 95% CI, 0.20-1.29; P=0.15; I^2^=0%). Four trials [32, 45, 75, 80] were included in the pooled RR for dizziness (RR, 0.94; 95% CI, 0.24-3.62; P=0.92; I^2^=0%). Four trials [42, 67, 70, 83] were included in the pooled RR for liver injury (RR, 1.40; 95% CI, 0.31-6.24; P=0.66; I^2^=0%). Two trials [67, 70] were included in the pooled RR for hypoglycemia (RR, 1.77; 95% CI, 0.39-8.04; P=0.46; I^2^=0%). One trial [87] reported three dropout cases due to hyperkalemia, of which two occurred in the treatment group and one in the control group. However, there was no statistically significant difference in the dropout rate due to hyperkalemia between study groups (RR, 2.00; 95% CI, 0.19-20.86; P=0.56). Coughing and hypotension were reported in one trial (RR, 2.84; 95% CI, 0.12-67.36; P=0.52) [67]. Nineteen [27, 33, 41, 43, 46, 52, 57, 58, 64–66, 69, 76, 79, 81, 82, 84, 88, 89] of 72 RCTs provided no data regarding ADEs despite clear descriptions of improvements in proteinuria, kidney function, and clinical symptoms. Effects of* A. manihot* on the likelihood of ADEs are shown in Table 2.

### 3.4. Strength of Evidence

The GRADE approach was used to assess the quality of the evidence and risk of bias. The results are shown in Table 3. The quality of evidence was generally low.

## 4. Discussion

### 4.1. Summary of Evidence

This is the first comprehensive systematic review and meta-analysis to assess the effects of* A. manihot* for DN patients with a diverse range of baseline protein level in urine and kidney function. None of the included trials reported the ESRD rate, and the pooled analysis of 7 trials indicated that there were no statistically significant differences between* A. manihot* plus a RAS blocker and a RAS blocker alone on eGFR. Thus, there was limited evidence to make a conclusion on the ESRD rate and eGFR. The results showed that compared to a RAS blocker, combined treatment of* A. manihot* and a RAS blocker was associated with significant improvement in proteinuria, UAER, and SCr, and the 24h UP reduction rate as well as normalization of UAER. The results also indicated that* A. manihot* might be generally well tolerated, because* A. manihot* added to a RAS blocker did not increase the rates of adverse events. However, due to the generally poor methodological quality and significant heterogeneity, there was currently insufficient evidence to support the routine use of* A. manihot* for DN. If confirmed in larger high-quality studies, these findings indicate that* A. manihot* might have an important role in improving proteinuria and protecting kidney function.

### 4.2. Limitations

Although this review is the most comprehensive meta-analysis to date regarding the safety and efficacy of* A. manihot* in combination with a RAS blocker for DN patients, there are limitations that should be considered.

Firstly, the methodological quality of these studies was generally low. Most described randomization poorly. None of the trials described allocation concealment and blinding. Only one [33] used a placebo control. One study [87] was given a grade of high risk for attrition bias (incomplete outcome data) due to the lack of information on how missing data were handled in the analysis. This meta-analysis carried the risk of reporting bias because not all studies reported all outcomes of interest. All the studies were single centered with generally small sample size, which might have resulted in the lack of power. Heterogeneity was significant among these studies, which weakened confidence in the results. Therefore, the results should be interpreted with caution due to the generally low methodological quality and significant heterogeneity.

Secondly, the study periods for all the identified studies were relatively short, resulting in the lack of evidence on the long-term effects of* A. manihot* for DN. In this systematic review, two studies [45, 91] reported that* A. manihot* was associated with a greater improvement in UAER after 8-week therapy, and the effect could persist for 12 weeks after treatment. However, most trials included assessed the short-term curative effect and did not continue with the follow-up to investigate the long-term effects of* A. manihot* on the prognosis of DN. Therefore, long-term studies are required to identify whether* A. manihot* could further reduce the rate of the ESRD.

Thirdly, close attention should be paid to ADEs. Safety is a fundamental principle for health care. Current evidence indicated that* A. manihot* combined with a RAS blocker might be relatively safe for DN. Nineteen of the included trials did not clearly provide data for ADEs despite all clear descriptions of great improvements in proteinuria or SCr with* A. manihot* therapy in this review. Future studies should pay special attention to ADEs of* A. manihot*.

### 4.3. Implication for Practice

DM is the most common cause of ESRD in the developed world [2]. In outcome trials of patients with DN, retrospective analyses demonstrate a robust relationship between magnitude of albuminuria reduction and slowing of CKD progression as well as reduced cardiovascular event rates [7–12]. The results indicated that* A. manihot *in addition to a RAS blocker seemed effective and safe, to reduce albuminuria further in patients with DN. However, due to the generally poor quality and significant heterogeneity, high-quality clinical studies are required to confirm these effects.

The main chemical constituents of* A. manihot* are flavonoids. Seven flavonoids, including hibifolin, hyperoside, myricetin, quercetin, isoquercetin, quercetin-3′-O-glucoside, and quercetin-3-O-robinobioside, were determined to be the major pharmacologically bioactive constituents of* A. manihot* by high-performance liquid chromatography (HPLC) [21, 99].* A. manihot* was shown to improve proteinuria, renal function, kidney inflammation, and glomerular injury and attenuate renal fibrosis, podocyte apoptosis, and mesangial proliferation. The renoprotective effects of* A. manihot* are related to inhibition of caspase-3 and caspase-8 overexpression, reduction of the ED1+ and ED3+ macrophages, attenuation of oxidative stress (OS), downregulation of the p38 mitogen-activated protein kinase (p38MAPK) and serine-threonine kinase (Akt) pathways, the suppression of transforming growth factor-*β*1 (TGF-*β*1) and tumor necrosis factor-a (TNF-*α*) protein expression, as well as the inhibition of the expression of *α*-smooth muscle actin, phosphorylation-extracellular signal-regulated kinase (p-ERK1/2), nicotinamide adenine dinucleotide phosphate (NADPH) Oxidase 1, NADPH Oxidase 2, and NADPH Oxidase 4 [100–103].

In this analysis, the results showed that* A. manihot* added to a RAS blocker could further improve proteinuria and kidney function in DN patients. Four previous meta-analyses [16–19] of* A. manihot* for DN preliminarily reported that* A. manihot* therapy showed great improvements in proteinuria and kidney function, which was consistent with this analysis. The review found that* A. manihot* for DN was well tolerated with minimal ADEs. Since the Huangkui capsule gained national approval from the China Food and Drug Administration in 1999, there have been no reports of severe ADEs. Previous meta-analyses [16–19] of* A. manihot* for DN reported that the most common adverse event was mild to moderate gastrointestinal discomfort; other ADEs such as dizziness, headache, and dry mouth were rarely reported. In this analysis, nine types of adverse events were observed, including gastrointestinal discomfort, dry mouth, headache, dizziness, liver injury, hypoglycemia, hyperkalemia, coughing, and hypotension. Well-tolerated gastrointestinal discomfort was still the most common ADE. Other side effects were not frequently reported. Rates of adverse events were not significantly different between the study groups except for headache, which was reported to occur more commonly in the control group. Although 19 of the included trials provided no data for ADEs, these studies all clearly reported that* A. manihot* was associated with significant improvements in proteinuria, SCr, and clinical symptoms. If confirmed, these results suggest that* A. manihot* might be effective and relatively safe for DN.

## 5. Conclusions

*A. manihot* in addition to a RAS blocker appeared to be effective and safe to further improve proteinuria and protect kidney function in patients with DN. However, due to the generally low methodological quality, significant heterogeneity, and publication bias of included articles, high-quality clinical studies are required to confirm these findings before the routine use of* A. manihot* can be recommended.

## Figures and Tables

**Table 1:** Characteristics of 72 included studies on *Abelmoschus manihot* for diabetic nephropathy.

Included studies	No. of patients	Mean age, y	Male, %	Baseline 24h UP (g/d)	Intervention		Duration (weeks)	Outcome measures
					Treatment group	Control group		
Chang LL2009[27]	128	47.2	61	3.7	Benazepril 10 mg/d + HK 3.0 g tid	Benazepril 10 mg/d	4	A
Chen XB 2014[28]	150	58.4	51	2.4	Benazepril 10 mg/d + HK 2.5 g tid	Benazepril 10 mg/d	8	AFG
Cheng Y 2016[29]	90	58.8	52	NA	Valsartan 80 mg/d + HK 2.5 g tid	Valsartan 80 mg/d	8	CEFG
Dai X 2017[30]	80	46.7	46	NA	Valsartan 80 mg/d + HK 2.5 g tid	Valsartan 80 mg/d	12	CFG
Deng SY 2014[31]	60	43.8	50	NA	Valsartan 80 mg/d + HK 2.5 g tid	Valsartan 80 mg/d	16	CFG
Du Y 2015[32]	73	59.4	45	1.0	ACEI/ARB + HK 2.5 g tid	ACEI/ARB	12	ADFG
Fan HW 2014[33]	52	49.1	48	NA	Candesartan 4 mg/d + HK 2.5 g tid	Candesartan 4 mg/d +placebo 2.5 g tid	24	CF
Gao Q 2017[34]	80	53.7	68	3.3	Irbesartan 150 mg/d + HK 2.5 g tid	Irbesartan 150 mg/d	16	ABCFG
Gu J 2015[35]	200	68.3	55	1.8	Valsartan 80 mg/d + HK 2.5 g tid	Valsartan 80 mg/d	8	ACDFG
Guan ZX 2008[36]	80	45.5	54	1.4	ACEI + HK 2.5 g tid	ACEI	8	AG
Guo G 2015[37]	136	42.8	53	0.3	Valsartan 80 mg/d + HK 2.5 g tid	Valsartan 80 mg/d	8	ABCFG
He YN 2010[38]	80	44.4	54	NA	Valsartan 80 mg/d + HK 2.5 g tid	Valsartan 80 mg/d	12	EFG
Hu JP 2011[39]	80	NA	NA	1.5	Telmisartan 80 mg/d + HK 2.5 g tid	Telmisartan 80 mg/d	8	AFG
Hu YY 2016[40]	40	56.9	60	0.1	Valsartan 80 mg/d + HK 2.5 g tid	Valsartan 80 mg/d	8	ADFG
Huang XM 2016[41]	82	43.4	57	0.4	Valsartan 80 mg/d + HK 2.5 g tid	Valsartan 80 mg/d	16	ABCF
Jia ZW 2015[42]	70	51.4	64	2.7	Candesartan 4 mg/d + HK 2.0 g tid	Candesartan 4 mg/d	4	AFG
Jiang ZJ 2012[43]	66	50.7	64	2.2	ACEI/ARB + HK 2.5 g tid	ACEI/ARB	16	AFD
Li HY 2009[44]	80	52.1	50	1.2	Fosinopril 10 mg/d + HK 2.5 g tid	Fosinopril 10 mg/d	8	AFG
Li QH 2010[45]	72	52.5	63	NA	Valsartan 80 mg/d + HK 2.5 g tid	Valsartan 80 mg/d	8	CG
Li WQ 2015[46]	72	48.3	57	0.3	Irbesartan 150 mg/d + HK 2.5 g tid	Irbesartan 150 mg/d	12	ACF
Li XM 2017[47]	62	60.9	54	NA	Valsartan 80 mg/d + HK 2.5 g tid	Valsartan 80 mg/d	8	CEG
Li YH 2016[48]	65	49.3	53	0.9	Benazepril 10 mg/d + HK 2.0 g tid	Benazepril 10 mg/d	16	ACFG
Li YL 2007[49]	60	41.5	54	1.5	Benazepril 10 mg/d + HK 2.5 g tid	Benazepril 10 mg/d	8	AFG
Li YL 2017[50]	86	59.2	52	NA	Valsartan 80 mg/d + HK 2.5 g tid	Valsartan 80 mg/d	8	CEG
Li YT 2014[51]	95	48.4	63	NA	Valsartan 80 mg/d + HK 2.5 g tid	Valsartan 80 mg/d	6	CFG
Li ZY 2014[52]	126	57.4	NA	0.4	ACEI/ARB + HK 2.5 g tid	ACEI/ARB	24	ACF
Liang F 2015[53]	100	44.8	54	NA	Valsartan 80 mg/d + HK 2.5 g tid	Valsartan 80 mg/d	6	CFG
Liang YP 2014[54]	50	43.2	47	NA	Valsartan 80 mg/d + HK 2.5 g tid	Valsartan 80 mg/d	8	CG
Liao YY 2017[55]	92	58.5	57	NA	Valsartan 80 mg/d + HK 2.5 g tid	Valsartan 80 mg/d	8	CG
Liu AY 2014[56]	100	60.5	43	2.4	Losartan Potassium 50 mg/d+HK 2.5 g tid	Losartan Potassium 50 mg/d	12	AFG
Liu H 2010[57]	80	NA^a^	54	6.2	Irbesartan + HK 2.5 g tid	Irbesartan	8	A
Liu JF 2011[58]	48	NA^a^	67	2.4	Candesartan + HK 2.5 g tid	Candesartan	12	AF
Lu C 2015[59]	80	42.1	54	NA	Valsartan 80 mg/d + HK 2.5 g tid	Valsartan 80 mg/d	8	CG
Luan R 2012[60]	96	42.3	59	3.8	ACEI/ARB + HK 2.5 g tid	ACEI/ARB	12	AFG
Ma F 2016[61]	80	49.1	54	NA	Valsartan 80 mg/d + HK 2.5 g tid	Valsartan 80 mg/d	6	CFG
Meng Y 2017[62]	86	45.9	56	NA	Valsartan 80 mg/d + HK 2.5 g tid	Valsartan 80 mg/d	8	CEG
Pan Q 2016[63]	96	64.6	65	0.7	Benazepril 10 mg/d or Valsartan 80 mg/d+ HK 2.5 g tid	Benazepril 10 mg/d or Valsartan 80 mg/d	8	ACFG
Qi MG 2016[64]	84	60.9	51	0.2	Valsartan 80 mg/d + HK 2.5 g tid	Valsartan 80 mg/d	12	AF
Qian C 2014[65]	120	51.0	53	2.4	Irbesartan 150 mg/d + HK 2.0 g tid	Irbesartan 150 mg/d	16	A
Qian CF 2010[66]	59	51.1	51	NA	Valsartan 80 mg/d + HK 2.5 g tid	Valsartan 80 mg/d	16	C
Qian JL 2013[67]	70	47.6	69	NA	Candesartan 4 mg/d + HK 2.5 g tid	Candesartan 4 mg/d	24	CFG
Qiao B 2015[68]	60	52.8	33	NA	Benazepril 10 mg/d + HK 2.5 g tid	Benazepril 10 mg/d	12	CFG
Qiao Y 2015[69]	82	56.9	63	2.4	Enalapril 10 mg/d + HK 2.5 g tid	Enalapril 10 mg/d	8	ADF
Qu XS 2013[70]	56	45.3	55	NA	Candesartan 4 mg/d + HK 2.5 g tid	Candesartan 4 mg/d	24	CFG
Rao WP 2016[71]	58	42.1	60	4.4	Benazepril 10 mg/d + HK 2.5 g tid	Benazepril 10 mg/d	8	AFG
Shen LL 2010[72]	82	42.3	61	NA	ACEI/ARB + HK 2.5 g tid	ACEI/ARB	12	FG
Song XL 2012[73]	60	40.7	NA	4.6	Benazepril + HK 2.5 g tid	Benazepril	12	AFG
Su JP 2009[74]	65	54.2	55	1.0	ACEI/ARB + HK 2.5 g tid	ACEI/ARB	24	ABCDEFG
Sun XM 2012[75]	90	62.3	63	3.7	Benazepril 10 mg/d + HK 2.5 g tid	Benazepril 10 mg/d	12	ADFG
Wang XC 2010[76]	63	59.2	51	0.4	Valsartan 80 mg/d + HK 2.5 g tid	Valsartan 80 mg/d	16	ACF
Wu RK 2017[77]	50	53.7	65	NA	Valsartan 80 mg/d + HK 2.5 g tid	Valsartan 80 mg/d	6	CEFG
Wu YH 2016[78]	48	56.1	54	1.5	Irbesartan 150-300 mg/d + HK 2.5 g tid	Irbesartan150-300 mg/d	16	AFG
Xiao ZZ 2010[79]	65	58.3	52	0.3	Valsartan 80 mg/d + HK 2.5 g tid	Valsartan 80 mg/d	16	ABCF
Xu GH 2014[80]	80	58.3	52	2.7	Telmisartan 80 mg/d + HK 2.5 g tid	Telmisartan 80 mg/d	12	ADFG
Xu RF 2012[81]	90	58.0	64	NA	Valsartan 80 mg/d + HK 2.5 g tid	Valsartan 80 mg/d	8	F
Xu SS 2016[82]	124	43.5	54	NA	Valsartan 80 mg/d + HK 2.5 g tid	Valsartan 80 mg/d	24	CF
Xu WM 2013[83]	61	36.0	56	NA	Irbesartan 150 mg/d + HK 2.5 g tid	Irbesartan 150 mg/d	4	CFG
Yan QJ 2015[84]	120	65.9	66	NA	Valsartan 80 mg/d + HK 2.5 g tid	Valsartan 80 mg/d	8	ACDF
Yan XP 2017[85]	70	52.9	47	NA	Valsartan 80 mg/d + HK 2.5 g tid	Valsartan 80 mg/d	10	EG
Yu JY 1995[86]	68	54.6	65	0.9	Captopril+Abelmoschus alcohol extraction 0.4 g tid	Captopril	8	ADFG
Yu ZW 2011[87]	58	69.0	45	1.5	Candesartan 8 mg/d + HK 2.5 g tid	Candesartan 8 mg/d	24	AFG
Zeng Y 2013[88]	50	67.6	54	2.3	Losartan 50 mg/d + HK 2.5 g tid	Losartan 50 mg/d	12	AF
Zhang H 2011[89]	58	58.2	55	1.7	Valsartan 80-160 mg/d + HK 2.5 g tid	Valsartan 80-160 mg/d	8	ACDEF
Zhang JW 2017[90]	112	53.0	48	NA	Valsartan 80 mg/d + HK 2.5 g tid	Valsartan 80 mg/d	16	CG
Zhang RX 2016[91]	80	51.3	53	NA	Valsartan 80 mg/d + HK 2.5 g tid	Valsartan 80 mg/d	8	CG
Zhang YS 2014[92]	110	50.0	56	3.4	Valsartan 80 mg/d + HK 2.5 g tid	Valsartan 80 mg/d	16	ABCFG
Zhang ZY 2017[93]	80	64.1	54	1.9	Valsartan 80 mg/d + HK 2.5 g tid	Valsartan 80 mg/d	8	ABFG
Zhao DH 2017[94]	80	50.8	54	NA	Valsartan 80 mg/d + HK 2.5 g tid	Valsartan 80 mg/d	6	CEFG
Zhao Y 2015[95]	92	49.2	55	NA	Valsartan 80 mg/d + HK 2.5 g tid	Valsartan 80 mg/d	8	CFG
Zhou BX 2008[96]	97	NA	NA	0.97	Benazepril 10 mg/d + HK 2.5 g tid	Benazepril 10 mg/d	8	ACG
Zhou XJ 2016[97]	96	66.1	59	NA	Valsartan 80 mg/d + HK 2.5 g tid	Valsartan 80 mg/d	8	CFG
Zhu JL 2017[98]	84	59.8	58	NA	Valsartan 80 mg/d + HK 2.5 g tid	Valsartan 80 mg/d	8	CEFG

Notes: HK: Huangkui Capsule, a single medicament of TCM extracted from the dry corolla of *Abelmoschus manihot*, acquired regulatory approval from China's State Food and Drug Administration (SFDA) for the treatment of chronic nephritis in 1999; ACEI: angiotensin-converting enzyme inhibitor; ARB: angiotensin receptor blocker; NA: not available; NA^a^: age range was reported, but mean age was not available; A: 24 h UP, 24-h urinary protein; B: eGFR: Estimated glomerular filtration rate; C: UAER, urinary albumin excretion rate; D: 24-h urinary protein (24 h UP) reduction rate, defined as the proportion of 24h UP decrease in protein excretion ≥50% of the baseline at the end of the study; E: normalization of urinary albumin excretion rate (UAER), defined as the proportion of UAER <20 *μ*g/min upon study completion; F: SCr, serum creatinine; G: ADEs, adverse drug events.

**Table 2:** Effect of *Abelmoschus manihot* on the likelihood of adverse drug events.

ADEs	No. of studies	Events		RR (95% CI)	*P*
		Treatment group (n/N)	Control group (n/N)		

Gastrointestinal discomfort	21	29/912	20/891	1.24 (0.72-2.13)	0.45
Dry mouth	11	5/528	16/527	0.51 (0.20-1.29)	0.15
Headache	10	1/520	16/515	0.29 (0.11-0.76)	0.01
Dizziness	4	4/164	4/151	0.94 (0.24-3.62)	0.92
Liver injury	4	4/135	2/122	1.40 (0.31-6.24)	0.66
Hypoglycemia	2	4/61	2/65	1.77 (0.39-8.04)	0.46
Hyperkalemia	1	2/29	1/29	2.00 (0.19-20.86)	0.56
Coughing	1	1/36	0/34∗	2.84 (0.12-67.36)	0.52
Hypotension	1	1/36	0/34∗	2.84 (0.12-67.36)	0.52
Total events	26	51/2421	61/2368	0.91 (0.63-1.31)	0.61

Notes: ADEs: adverse drug events; CI, confidence interval; RR, risk ratio; ∗: a standard correction of 0.5 was added to all cells when a 0 cell existed in a 2X2 table for the calculation of RR.

**Table 3:** GRADE Evidence Profile for *Abelmoschus manihot* in addition to a renin-angiotensin system blocker for diabetic nephropathy.

Outcome	No. of studies	No. of participants	Quality assessment					Summary of findings	
			Risk of bias	Inconsistency	Indirectness	Imprecision	Publication bias	Effect size (95% CI)	Quality

24h UP	41	3464	Serious^1^	Serious^2^	No serious indirectness	No serious imprecision	Reporting bias^3^	MD, -0.39(-0.46 to -0.33)	+, Very low
UAER	42	3544	Serious^1^	Serious^2^	No serious indirectness	No serious imprecision	Reporting bias^3^	MD, -19.90( -22.62 to -17.18)	+, Very low
24h UP reduction rate	11	948	Serious^1^	No serious inconsistency	No serious indirectness	No serious imprecision	None	RR, 1.43(1.26 to 1.63)	+++, Moderate
Normalization of UAER	11	811	Serious^1^	No serious inconsistency	No serious indirectness	No serious imprecision	None	RR, 1.48(1.29 to 1.70)	+++, Moderate
SCr	56	4541	Serious^1^	Serious^2^	No serious indirectness	No serious imprecision	Reporting bias^3^	MD, -7.35( -9.95 to -4.76)	+, Very low

Notes: GRADE, Grades of Recommendation, Assessment, Development and Evaluation; RR, risk ratio; MD, mean difference; CI, confidence interval; 24h UP, 24-h urinary protein; UAER, urinary albumin excretion rate; SCr, serum creatinine; ^1^unclear allocation concealment in all studies; ^2^meta-analysis for the outcome exhibited significant heterogeneity; ^3^the funnel plot was asymmetrical, indicating a potential publication bias.

## Data Availability

All relevant data are within the paper and its supporting information files.

## Supplementary Materials

Supplementary 1 File S1: PRISMA Checklist of Abelmoschus manihot for diabetic nephropathy.

Supplementary 2 File S2: PubMed search strategy.
