# Supplementary material for: Abelmoschus manihot for Diabetic Nephropathy: A Systematic Review and Meta-Analysis
Source: Evid Based Complement Alternat Med. 2019 Apr 18;2019:9679234. doi: 10.1155/2019/9679234 (PMC6500631; doi:10.1155/2019/9679234)
Supplement: Supplementary 1 — File S1: PRISMA Checklist of Abelmoschus manihot for diabetic nephropathy. [file 9679234.f1.doc]

| **Section/topic** | **#** | **Checklist item** | **Reported on page #** |
| --- | --- | --- | --- |
| **TITLE** | | |  |
| Title | 1 | Abelmoschus manihot for diabetic nephropathy: a systematic review and meta-analysis of 72 randomized controlled trials | 1 |
| **ABSTRACT** | | |  |
| Structured summary | 2 | **Background**Diabetic nephropathy (DN) is the leading cause of end-stage renal disease. Present medications for DN are not satisfactory. Many trials showed that Abelmoschus manihot, a single medicament of traditional Chinese medicine, when added to a renin-angiotensin system (RAS) blocker, could further reduce proteinuria and protect kidney function in patients with DN. It is essential to systematically assess the efficacy and safety of Abelmoschus manihot for DN.**Methods**Eight English and Chinese electronic databases were searched, from inception to December 2017, to identify randomized controlled trials (RCTs) of Abelmoschus manihot for DN. The Cochrane Risk of Bias tool was used to evaluate the methodological quality of eligible studies. Meta-analysis was performed by Review Manager 5.3.**Results**Seventy-two RCTs with 5895 participants were identified. The methodological quality of the included studies was generally low. The meta-analysis indicated that compared to a RAS blocker, combined treatment by Abelmoschus manihot and a RAS blocker was more effective in terms of the 24h urinary protein (24h UP) (MD, -0.39[95% CI, -0.46 to -0.33] g/d; P<0.00001), urinary albumin excretion rate(UAER)(MD, -19.90[95% CI, -22.62 to -17.18]ug/min; P<0.00001), 24h UP reduction rate (RR, 1.43; 95% CI, 1.26-1.63; P<0.00001) and normalization of UAER (RR, 1.48; 95%CI, 1.29-1.70; P<0.00001), as well as serum creatinine (Scr) (MD, -7.35[95% CI, -9.95 to -4.76] umol/L; P <0.00001). None of the included trials reported rate of ESRD. There were no statistically significant differences between Abelmoschus manihot plus a RAS blocker and a RAS blocker alone on estimated glomerular filtration rate (eGFR)(MD, 4.43 [95% CI, -1.68 to 10.54]mL/min; P=0.16). Abelmoschus manihot did not increase the rates of adverse drug events or reactions.**Conclusions**Abelmoschus manihot in addition to a RAS blocker appeared to be effective and safe to further reduce proteinuria and protect kidney function in patients with DN. However, due to the generally low methodological quality, significant heterogeneity and publication bias, high-quality RCTs are required to confirm these findings before the routine use of Abelmoschus manihot can be recommended.**Keywords**: Abelmoschus manihot, Diabetic kidney disease, Systematic review, Meta-analysis | 2-3 |
| **INTRODUCTION** | | |  |
| Rationale | 3 | Type 2 diabetes mellitus (T2DM) is the most common cause of chronic kidney disease (CKD) and end-stage renal disease (ESRD) in the developed world, and the second leading cause of ESRD after primary glomerular disease in China. DM and CKD are independent risk factors for all-cause mortality as well as cardiovascular death. DN poses the highest risk for death compared to DM or CKD alone. The effectiveness of current interventions for DN is still limited given the number of patients that continue to have progression of their renal dysfunction despite the use of existing renin-angiotensin system (RAS) blockers. Retrospective analyses of clinical studies concerning DN demonstrate a strong relationship between magnitude of albuminuria reduction and slowing of DN progression as well as reduced cardiovascular event rates. An angiotensin-converting enzyme inhibitor (ACEI) combined with an angiotensin receptor blocker(ARB) is not recommended due to high risk of hyperkalemia and/ or acute kidney injury as well as for futility. Recently, concomitant mineralocorticoid receptor antagonist (MRA) has been studied as a novel approach to further prevent the progression of DN. A meta-analysis by Mavrakanas TA et al reported that combined treatment by an ACEI/ARB and a MRA was effective in decreasing albuminuria compared to standard treatment by an ACEI/ARB in DN but increased the risk of hyperkalemia. Therefore, there is an urgent need for a new pharmacologic agent that could be effective and safe to further reduce proteinuria and prevent the progression of DN.Abelmoschus manihot has been used for chronic glomerulonephritis in China for centuries. Huangkui capsule, a single medicament of TCM extracted from the dry corolla of A manihot, acquired regulatory approval from China's State Food and Drug Administration (SFDA) for the treatment of chronic nephritis in 1999. A manihot can ameliorate proteinuria and improve kidney function in patients with CKD, including DN, immunoglobulin A nephropathy (IgAN), membranous nephropathy, which is currently considered as an important adjuvant therapy for CKD. The major biologically active constituents are total flavones of A manihot (TFA). Mechanistic studies applying A manihot to the treatment of CKD suggest that the major effects are associated with improved immunological reaction, inflammation, renal fibrosis, and renal tubular epithelial injury. The results of previous meta-analyses preliminarily suggested that A manihot could reduce proteinuria and protect kidney function in patients with DN. However, the evidence was very limited on the effect of A manihot for DN due to a limited number of included trials with poor methodological quality. A lot of novel data evaluating A manihot in DN have been recently published. Therefore, we systematically analyzed the evidence on A manihot therapy as an adjuvant to a RAS blocker in DN, focusing on its effect in albuminuria. | 3-4 |
| Objectives | 4 | To assess systematically and critically the efficacy and safety of Abelmoschus manihot in addition to a renin-angiotensin system (RAS) blocker for diabetic kidney disease. | 4 |
| **METHODS** | | |  |
| Protocol and registration | 5 | The review protocol was registered with the International Prospective Register of Systematic Reviews (PROSPERO registration no.CRD42018087182, available from: http://www.crd.york.ac.uk/PROSPERO/display_record.php?ID= CRD42018087182, additional file 2). | 4-5(S2 File) |
| Eligibility criteria | 6 | **Inclusion criteria**: Studies were included if they met the following criteria: (1) randomized controlled design; (2) patients with type 1 or type 2 diabetes mellitus and diabetic nephropathy (defined as at least 30 mg of albuminuria in a 24-h urine collection or urinary albumin excretion rate (UAER) of at least 20ug/min); (3)participants should have received an ACEI or an ARB throughout the study as standard treatment. To evaluate the effect of concomitant A manihot, a subset of patients in each study should also receive A manihot in addition to standard RAS blockade;(4)the primary outcome measures included 24h urinary protein (24h UP), rate of ESRD and estimated glomerular filtration rate (eGFR). The secondary outcome measures were UAER, improvements in 24h UP reduction rate(defined as a proportion of 24h UP decreasing to protein excretion≥50% of baseline at the end of the study), normalization of UAER(defined as a proportion of UAER of less than 20 ug/min upon study completion), serum creatinine (Scr) and adverse drug events (ADEs) or adverse drug reactions (ADRs) during the scheduled treatment and follow-up. (5) the studies contained available and relevant data for meta-analysis; (6)the studies were available in any language.**Exclusion criteria**: Excluded from the meta-analysis were duplicated publications, studies with unavailable or incorrect data, articles not reporting outcomes of interest. Also excluded were studies enrolling fewer than 10 participants, quasi-randomized controlled trials (that is, allocation using alternation, the sequence of admission, case record numbers, dates of birth), and non-randomized controlled clinical trials. Studies using combination RAS blockers as background therapy or A manihot combined with any other herbal medicines were excluded to avoid confounding information. | 5-6 |
| Information sources | 7 | The Cochrane Central Register of Controlled Trials (CENTRAL) on the Cochrane Library, PubMed, EMBASE, Chinese National Knowledge Infrastructure database (CNKI), Chinese Biomedical Literature database (CBM), Chinese Scientific Journal database (VIP), and Wan Fang database were searched to identify eligible trials published from inception to December 15, 2017. Ongoing registered clinical trials were searched in the Clinical Trials, gov (https://www. clinical trials.gov/). The articles were not restricted based on language. All included studies were subjected to the same quality assessment. The following search terms were used: Abelmoschus, Abelmoschus manihot, Flos Abelmoschus manihot, Abelmoschus corolla, Abelmoschus moschatus Medicus, okra, ambrette, Huangkui, Huangkui capsule, huangshukui, huangshukuihua, diabetic nephropathy, diabetic nephropathies, DN, diabetes mellitus, diabetes, diabetic, kidney disease, renal disease, diabetic kidney disease, DKD, diabetic renal disease, albuminuria, randomized controlled trial, controlled clinical trial, randomized, placebo, drug therapy, randomly, trial, groups. An example of the full electronic search strategy was included in the Additional file 3. The literature search was performed independently by two authors (LW Shi and MZ Zhang), and disagreements were resolved by mutual discussion. The details for the full search strategy were listed in a flow diagram. | 4-5 |
| Search | 8 | **Four English electronic databases:** Cochrane Central Register of Controlled Trials (CENTRAL) on the Cochrane Library, PubMed, EMBASE, the Clinical Trials, gov.  **Four Chinese electronic databases:** Chinese National Knowledge Infrastructure(CNKI), Chinese Biomedical Literature Database (CBM), Chinese Scientific Journal Database (VIP), Wan Fang Database. | 4(additional file 3) |
| Study selection | 9 | Two authors (LW Shi and MZ Zhang) independently conducted the literature searching and study selection. | 4-5 |
| Data collection process | 10 | Two authors (XW Li and YN Yang) independently extracted information on patients, methods, interventions, outcomes and results using a predesigned data extraction form. The data extraction form included the following items: (1) general information: title, authors, year of publication; (2)trial characteristics: design, method of randomization, allocation concealment, incomplete outcome data, selective reporting, blinding (patients, people administering treatment, outcome assessors); (3)intervention(s): intervention (s) and comparison intervention(s) (dose, usage, duration and frequency); (4) patients: total number and number in both groups, baseline characteristics, dropouts and losses to follow up (reasons, description); (5)outcomes: outcomes specified above, quality of reporting of outcomes. Any disagreement was settled by mutual discussion or by consulting a third author (Q Ni). | 5-6 |
| Data items | 11 | **The primary outcome measures:** 24h urinary protein (24h UP) , rate of ESRD and estimated glomerular filtration rate (eGFR).  **The secondary outcome measures:** urinary albumin excretion rate (UAER), improvements in 24h urinary protein (24h UP) reduction rate(defined as a proportion of 24h UP decreasing to protein excretion ≥50% of baseline at the end of the study), normalization of UAER(defined as a proportion of UAER of less than 20 ug/min upon study completion), serum creatinine (Scr) and adverse drug events (ADEs) or adverse drug reactions (ADRs) during the scheduled treatment and follow-up. | 5-7(Table1) |
| Risk of bias in individual studies | 12 | The methodological quality of randomized controlled trials (RCTs) was assessed using each item specified by the Cochrane Risk of Bias Tool, including random sequence generation, allocation concealment, blinding of participants and personnel, blinding of outcome assessment, incomplete outcome date, selective reporting and other bias. Each study was respectively categorized as “low risk of bias”(all the items were in low risk), “high risk of bias”(one or more items were in high risk), “unclear risk of bias”(one or more items were in unclear risk). Low risk of bias represented a good quality. Authors were contacted by E-mail to obtain further data and verify methodological quality when necessary. The Grading of Recommendations Assessment, Development and Evaluation (GRADE) methodology was used to assess the quality of the evidence for each outcome. Any disagreement was settled by discussion or by consulting a third author (Q Ni). | 6 |
| Summary measures | 13 | Dichotomous outcomes were presented as risk ratio (RR) and 95% confidence intervals (CIs) and continuous outcomes were presented as mean difference (MD, defined as the difference between study groups at the end of study) and 95% CIs. | 6 |
| Synthesis of results | 14 | Meta-analysis was performed by RevMan 5.3. Dichotomous outcomes were presented as risk ratio (RR) and 95% confidence intervals (CIs) and continuous outcomes were presented as mean difference (MD, defined as the difference between study groups at the end of study) and 95% CIs. A random-effects model was used to pool the data. Statistical heterogeneity was assessed with the I-square (I^2^) statistic. The I^2^ statistic of 50% or less referred to low statistical heterogeneity, and that of more than 50% was considered substantial statistical heterogeneity. | 6-7 |

Page 1 of 2

| **Section/topic** | **#** | **Checklist item** | **Reported on page #** |
| --- | --- | --- | --- |
| Risk of bias across studies | 15 | Publication bias was assessed using funnel plots if the group included more than 10 trials. | 6-7 |
| Additional analyses | 16 | Sensitivity analysis was performed by excluding low-quality studies. | 6-7 |
| **RESULTS** | | |  |
| Study selection | 17 | A flow diagram of study selection was shown in Figure 1. During initial electronic search, 1,114 articles were identified, of which 962 were excluded including duplicates and irrelevant studies. The full texts of the selected 152 trials were retrieved, and after detailed evaluation, 72 RCTs[27-98] were finally selected for meta-analysis, of which 28 meeting the inclusion criteria from four previous meta-analyses[16-19]were included. Authors were contacted by E-mail for additional outcome data, however, no reply was received. | 6-7  (Figure 1) |
| Study characteristics | 18 | The baseline characteristics of DN patients are presented in additional file 3. 72 studies included a total of 5,895 patients followed up from 4 to 24 weeks. The treatment group consisted of 3,000 patients, the control group was comprised of 2,895 patients. The mean age reported for participants in these studies ranged from 36 to 69 years and the proportion of males ranged from 33.3% to 69.2%. The average baseline proteinuria was 1.94g/d (0.14-6.2)g/d. The median follow-up for 24h UP was 12 weeks. Baseline characteristics of the experimental and control groups were similar in all studies. All the included trials compared A manihot plus a RAS blocker to the RAS blocker alone. A manihot in the form of a Huangkui capsule, was given orally at 3.0g 3 times per day in one trial[27], given orally at 2.5g 3 times per day in 67 trials[28-41,43-47,49-64,66-85,87-98], given orally at 2.0g 3 times per day in three trials[42,48,65]. In one study[86], Abelmoschus alcohol extraction was given orally at 0.4g 3 times per day. A range of RAS blockers were used: 13(18.06%) studies[27,28,36,44,48,49,68,69,71,73,75,86,96]used ACEI (Captopril, Enalapril Maleate, Fosinopril, and Benazepril), 52 (72.22%) studies [29-31,33-35,37-42,45-47,50,51,53-59,61,62, 64-67,70,76-85,87-95,97,98]used ARB (Valsartan, Telmisartan, Candesartan, Irbesartan, and Losartan), 7(9.72%) studies[32,43,52,60,63,72,74] reported that ACEI or ARB was used. All other concomitant therapies, including insulin, oral antidiabetic drugs, antihypertensive drugs, and lipid-lowering drugs, were comparable between study groups. Trials were all single-center studies with a sample size that ranged from 40 to 200 and were published in 1995-2017. All the included trials were conducted in China, and published in Chinese. | 7(Table 1) |
| Risk of bias within studies | 19 | A summary of study quality was presented in Figure 2. The methodological quality of the included trials was generally poor. All trials were reported to be randomized, but only 14 (19.44%) trials [28,30,35,45,47,48,52,61,65,68,75,82,88, 94]described adequate sequence generation. Methods for allocation concealment were unclear in all studies. Blinding of participants and personnel nor blinding of outcome assessment were not mentioned in all trials. Risk of attrition bias (incomplete outcome data) was detected in one[87]of all included trials, earning the status of high risk. Selective reporting was generally unclear due to the inaccessibility to the trial protocol. Other potential sources of bias were unclear. The funnel plots based on 24h UP, UAER, Scr were asymmetrical, indicating that potential publication bias might affect the results of this meta-analysis. The funnel plots constructed for improvements in 24h UP reduction rate and normalization of UAER, both were nearly symmetrical, indicating that potential publication bias might not affect the results of this meta-analysis. Funnel plots based on the outcomes were elaborated in Figure 3. | 7  (Figure 2, Figure3) |
| Results of individual studies | 20 | **Primary outcomes**  **24-h urinary protein (24h UP)**  Data regarding the effect of A manihot combined with a RAS blocker compared to a RAS blocker on 24h UP were available from 41[27,28,32,34-37,39-44,46,48,49,52,56-58,60,63-65,69,71,73-76,78-80,84,86-89,92,93, 96] of 72 trials, including 3,464 participants. The meta-analysis indicated that A manihot plus a RAS blocker was associated with significant reductions in 24h UP level compared with a RAS blocker alone at the end of study(MD, -0.39[95% CI, -0.46 to -0.33] g/d; P<0.00001; Figure 4). There was evidence of significant heterogeneity across these trials (I2 =98%; P for heterogeneity < 0.00001; Figure 4).  **End-stage renal disease (ESRD) and estimated glomerular filtration rate (eGFR)**  None of the included trials performed the assessment of rate of ESRD. Seven trials[34,37,41,74,79,92,93] with 618 patients assessed the effect of A manihot plus a RAS blocker on eGFR in patients with DN. The pooled analysis of 7 trials indicated that there were no statistically significant differences between A manihot plus a RAS blocker and a RAS blocker alone on eGFR(MD, 4.43 [95% CI, -1.68 to 10.54]mL/min; P=0.16; Figure 5). There was evidence of significant heterogeneity across these trials (I2 =89%; P for heterogeneity < 0.00001; Figure 5).  **Secondary outcomes**  **Effect on urinary albumin excretion rate (UAER)**  The effect of A manihot combined with a RAS blocker versus a RAS blocker on UAER level was reported in 42 trials[29-31,33-35,37,41,45-48,50-55,59,61-63,66-68,70,74,76,77,79,82-84,89-92,94-98], including 3,544 participants. The meta-analysis indicated that compared to a RAS blocker alone, A manihot combined with a RAS blocker was associated with a greater decrease in UAER (MD, -19.90 [95% CI, -22.62 to -17.18]ug/min; P<0.00001; Figure 6). Again, there was evidence of significant heterogeneity across these trials (I2 =99%; P for heterogeneity< 0.00001; Figure 6). In addition, two studies[45,91] of 42 trials reported that trial duration per patient was 20 weeks, with 8 weeks of treatment and 12 weeks of follow-up without treatment. The mean difference of UAER between study groups at the end of follow-up was assessed again and was still less in treatment group versus control group (one trial[45]: MD, -33.00[95 % CI, -42.93 to -23.07] ug/min; p<0.00001, and the other one[91]: MD, -11.40[95% CI, -14.91 to -7.89]ug/min; p<0.00001), indicating that the effect of A manihot on UAER might persist for 12 weeks after treatment.  **Improvements in 24h UP reduction rate and normalization of UAER**  Eleven[32,35,40,43,69,74,75,80,84,86,89]of the included studies reported changes in 24h UP reduction rate. The pooled results showed that A manihot combined with a RAS blocker therapy was associated with significant improvements in 24h UP reduction rate compared to a RAS blocker alone (RR, 1.43; 95% CI, 1.26-1.63; P<0.00001; Figure 7). The normalization of UAER was reported in 11 trials of 72 RCTs[29,38,47,50,62,74,77,85,89,94,98]. The meta-analysis indicated that combined treatment by A manihot and a RAS blocker was more effective in normalization of UAER (RR, 1.48; 95% CI, 1.29-1.70; P<0.00001; Figure 8). Statistical heterogeneity was low for these outcomes, suggesting a consistent effect size across studies (I2 =0%; Figure 7, 8).  **Serum creatinine(Scr)**  Data for the effect of A manihot combined with a RAS blocker compared to a RAS blocker on Scr level were available from 56 trials[28-35,37-44,46,48,49,51-53,56,58,60,61,63,64,67-84,86-89,92-95,97,98], including 4,541 participants. The meta-analysis indicated that compared with a RAS blocker alone, A manihot combined with a RAS blocker led to a greater decrease in Scr (MD, -7.35[95 % CI, -9.95 to -4.76] umol/L; P <0.00001, Figure 9), indicating that A manihot plus a RAS blocker was associated with improved kidney function. The I-square (I2) statistic based on the data for Scr exhibited significant heterogeneity among these trials (I2 = 89 %, P <0.00001, Figure 9).  **Adverse drug events or reactions (ADEs or ADRs)**  ADEs or ADRs were observed by 53 trials[28-32,34-40,42,44,45,47-51,53-56,59-63,67,68,70-75,77,78,80,83,85-87, 90-98] of 72 RCTs, of which 27 trials[28,34,36-40,47-50,55,56,62,63,68,71,73,74,77,85,86,92-94,96,98]reported that no ADEs or ADRs occurred in their trials, 26 trials[29-32,35,42,44,45,51,53,54,59-61,67,70,72,75,78,80,83,87,90,91, 95,97]reported that ADEs or ADRs occurred in their trials, including gastrointestinal discomfort, dry mouth, headache, dizzy, liver injury, hypoglycemia, hyperkalemia, coughing, and hypotension. There were no statistically significant differences between study groups in all rates of ADEs or ADRs except for headache, which was reported in 10 trials[29,35,51,53,61,75,80,90,95,97] and occurred more commonly in control group (RR, 0.29; 95%CI, 0.11-0.76; P=0.01; I2=0%). 21 trials[29-32,35,44,45,51,53,59-61,67,70,72, 78,83,90,91,95,97]were included in the pooled RR for gastrointestinal discomfort(RR, 1.24; 95%CI, 0.72-2.13; P=0.45; I2=0%). 11 trials [29,31,35,51,53,54,59,61,90,95, 97]were included in the pooled RR for dry mouth (RR, 0.51; 95%CI, 0.20-1.29; P=0.15;I2=0%). Four trials[32,45,75,80] were included in the pooled RR for dizzy(RR, 0.94; 95%CI, 0.24-3.62; P=0.92; I2=0%). Four trials[42,67,70,83]were included in the pooled RR for liver injury (RR,1.40; 95%CI, 0.31-6.24; P=0.66; I2=0%). Two trials[67,70] were included in the pooled RR for hypoglycemia (RR, 1.77; 95% CI, 0.39-8.04; P=0.46; I2=0%). One trial[87]reported three dropout cases due to hyperkalemia, of which two occurred in treatment group and one occurred in control group. However, there was no statistically significant difference in the dropout rate due to hyperkalemia between study groups (RR, 2.00; 95% CI, 0.19-20.86; P=0.56). Coughing and hypotension was reported in one trial[67](RR, 2.84; 95% CI, 0.12-67.36; P=0.52). 19 trials[27,33,41,43,46,52,57,58,64-66,69,76,79,81,82,84,88,89] of 72 RCTs provided no data regarding ADEs or ADRs despite clear descriptions of improvements in proteinuria, kidney function and clinical symptoms. Effects of A manihot plus a RAS blocker versus a RAS blocker on likelihood of ADEs or ADRs were shown in Table 2. | 8-10  (Figures 4-9, Table 2) |
| Synthesis of results | 21 | 1. **h urinary protein (24h UP):** Data regarding the effect of A manihot combined with a RAS blocker compared to a RAS blocker on 24h UP were available from 41[27,28,32,34-37,39-44,46,48,49,52,56-58,60,63-65,69,71,73-76, 78-80,84,86-89,92,93,96] of 72 trials, including 3,464 participants. The meta-analysis indicated that A manihot plus a RAS blocker was associated with significant reductions in 24h UP level compared with a RAS blocker alone at the end of study(MD, -0.39[95% CI, -0.46 to -0.33] g/d; P<0.00001; Figure 4). There was evidence of significant heterogeneity across these trials (I2 =98%; P for heterogeneity < 0.00001; Figure 4).   **End-stage renal disease (ESRD) and estimated glomerular filtration rate (eGFR)**  None of the included trials performed the assessment of rate of ESRD. Seven trials[34,37,41,74,79,92,93] with 618 patients assessed the effect of A manihot plus a RAS blocker on eGFR in patients with DN. The pooled analysis of 7 trials indicated that there were no statistically significant differences between A manihot plus a RAS blocker and a RAS blocker alone on eGFR(MD, 4.43 [95% CI, -1.68 to 10.54]mL/min; P=0.16; Figure 5). There was evidence of significant heterogeneity across these trials (I2 =89%; P for heterogeneity < 0.00001; Figure 5).  **Effect on urinary albumin excretion rate (UAER)**  The effect of A manihot combined with a RAS blocker versus a RAS blocker on UAER level was reported in 42 trials[29-31,33-35,37,41,45-48,50-55,59,61-63,66-68,70,74,76,77,79,82-84,89-92,94-98], including 3,544 participants. The meta-analysis indicated that compared to a RAS blocker alone, A manihot combined with a RAS blocker was associated with a greater decrease in UAER (MD, -19.90 [95% CI, -22.62 to -17.18]ug/min; P<0.00001; Figure 6). Again, there was evidence of significant heterogeneity across these trials (I2 =99%; P for heterogeneity< 0.00001; Figure 6). In addition, two studies[45,91] of 42 trials reported that trial duration per patient was 20 weeks, with 8 weeks of treatment and 12 weeks of follow-up without treatment. The mean difference of UAER between study groups at the end of follow-up was assessed again and was still less in treatment group versus control group (one trial[45]: MD, -33.00[95 % CI, -42.93 to -23.07] ug/min; p<0.00001, and the other one[91]: MD, -11.40[95% CI, -14.91 to -7.89]ug/min; p<0.00001), indicating that the effect of A manihot on UAER might persist for 12 weeks after treatment.  **Improvements in 24h UP reduction rate and normalization of UAER**  Eleven[32,35,40,43,69,74,75,80,84,86,89]of the included studies reported changes in 24h UP reduction rate. The pooled results showed that A manihot combined with a RAS blocker therapy was associated with significant improvements in 24h UP reduction rate compared to a RAS blocker alone (RR, 1.43; 95% CI, 1.26-1.63; P<0.00001; Figure 7). The normalization of UAER was reported in 11 trials of 72 RCTs[29,38,47,50,62,74,77,85,89,94,98]. The meta-analysis indicated that combined treatment by A manihot and a RAS blocker was more effective in normalization of UAER (RR, 1.48; 95% CI, 1.29-1.70; P<0.00001; Figure 8). Statistical heterogeneity was low for these outcomes, suggesting a consistent effect size across studies (I2 =0%; Figure 7, 8).  **Serum creatinine(Scr)**  Data for the effect of A manihot combined with a RAS blocker compared to a RAS blocker on Scr level were available from 56 trials[28-35,37-44,46,48,49,51-53,56,58,60,61,63,64,67-84,86-89,92-95,97,98], including 4,541 participants. The meta-analysis indicated that compared with a RAS blocker alone, A manihot combined with a RAS blocker led to a greater decrease in Scr (MD, -7.35[95 % CI, -9.95 to -4.76] umol/L; P <0.00001, Figure 9), indicating that A manihot plus a RAS blocker was associated with improved kidney function. The I-square (I2) statistic based on the data for Scr exhibited significant heterogeneity among these trials (I2 = 89 %, P <0.00001, Figure 9).  **Adverse drug events or reactions (ADEs or ADRs)**  ADEs or ADRs were observed by 53 trials[28-32,34-40,42,44,45,47-51,53-56,59-63,67,68,70-75,77,78,80,83,85-87, 90-98] of 72 RCTs, of which 27 trials[28,34,36-40,47-50,55,56,62,63,68,71,73,74,77,85,86,92-94,96,98]reported that no ADEs or ADRs occurred in their trials, 26 trials[29-32,35,42,44,45,51,53,54,59-61,67,70,72,75,78,80,83,87,90,91, 95,97]reported that ADEs or ADRs occurred in their trials, including gastrointestinal discomfort, dry mouth, headache, dizzy, liver injury, hypoglycemia, hyperkalemia, coughing, and hypotension. There were no statistically significant differences between study groups in all rates of ADEs or ADRs except for headache, which was reported in 10 trials[29,35,51,53,61,75,80,90,95,97] and occurred more commonly in control group (RR, 0.29; 95%CI, 0.11-0.76; P=0.01; I2=0%). 21 trials[29-32,35,44,45,51,53,59-61,67,70,72, 78,83,90,91,95,97]were included in the pooled RR for gastrointestinal discomfort(RR, 1.24; 95%CI, 0.72-2.13; P=0.45; I2=0%). 11 trials [29,31,35,51,53,54,59,61,90,95, 97]were included in the pooled RR for dry mouth (RR, 0.51; 95%CI, 0.20-1.29; P=0.15;I2=0%). Four trials[32,45,75,80] were included in the pooled RR for dizzy(RR, 0.94; 95%CI, 0.24-3.62; P=0.92; I2=0%). Four trials[42,67,70,83]were included in the pooled RR for liver injury (RR,1.40; 95%CI, 0.31-6.24; P=0.66; I2=0%). Two trials[67,70] were included in the pooled RR for hypoglycemia (RR, 1.77; 95% CI, 0.39-8.04; P=0.46; I2=0%). One trial[87]reported three dropout cases due to hyperkalemia, of which two occurred in treatment group and one occurred in control group. However, there was no statistically significant difference in the dropout rate due to hyperkalemia between study groups (RR, 2.00; 95% CI, 0.19-20.86; P=0.56). Coughing and hypotension was reported in one trial[67](RR, 2.84; 95% CI, 0.12-67.36; P=0.52). 19 trials[27,33,41,43,46,52,57,58,64-66,69,76,79,81,82,84,88,89] of 72 RCTs provided no data regarding ADEs or ADRs despite clear descriptions of improvements in proteinuria, kidney function and clinical symptoms. Effects of A manihot plus a RAS blocker versus a RAS blocker on likelihood of ADEs or ADRs were shown in Table 2. | 8-10  (Figures  4-9, Table 2) |
| Risk of bias across studies | 22 | The funnel plots based on 24h UP, UAER and Scr were asymmetrical, indicating that potential publication bias might affect the results of this meta-analysis. The funnel plots constructed for improvements in 24h UP reduction rate and normalization of UAER, both were nearly symmetrical, indicating that potential publication bias might not affect the results of this meta-analysis. | 7(Figure 3) |
| Additional analysis | 23 | Sensitivity analysis was not performed due to all included trials with generally low methodological quality. | 7 |
| **DISCUSSION** | | |  |
| Summary of evidence | 24 | **Summary of evidence**  This is the first comprehensive systematic review and meta-analysis to assess the effects of A manihot for DN patients with a diverse range of baseline proteinuria and kidney function. None of the included trials reported rate of ESRD, and the pooled analysis of 7 trials indicated that there were no statistically significant differences between A manihot plus a RAS blocker and a RAS blocker alone on eGFR. Thus evidence was limited to make a conclusion on rate of ESRD and eGFR. The results showed that compared to a RAS blocker, combined treatment by A manihot and a RAS blocker was associated with significant reductions in proteinuria, UAER, Scr, and significant improvements in 24h UP reduction rate as well as normalization of UAER. The results also indicated that A manihot might be generally well tolerated, because A manihot added to a RAS blocker did not increase the rates of adverse events. However, due to generally poor methodological quality, significant heterogeneity and publication bias, there was currently no enough evidence to support the routine use of A manihot for DN. If confirmed in larger high-quality studies, these results suggested that A manihot might have a role in reducing proteinuria and preventing the progression of DN.  **Implication for practice**  Diabetes mellitus is the most common cause of ESRD in the developed world. In outcome trials of patients with DN, retrospective analyses demonstrate a robust relationship between magnitude of albuminuria reduction and slowing of CKD progression as well as reduced cardiovascular event rates. The results indicated that A manihot in addition to a RAS blocker seemed effective and safe to reduce albuminuria further in patients with DN. However, due to generally poor methodological quality, significant heterogeneity and publication bias, high-quality RCTs are required to confirm these findings before the routine use of A manihot can be recommended.  The main chemical constituents of A manihot are flavonoids. Seven flavonoids, including hibifolin, hyperoside, myricetin, quercetin, isoquercetin, quercetin-3′-O-glucoside and quercetin-3-O-robinobioside, were determined to be the major pharmacologically bioactive constituents of A manihot by high-performance liquid chromatography (HPLC)[99,100]. A manihot has shown to reduce proteinuria, improve renal function, kidney inflammation and glomerular injury, attenuate renal fibrosis, podocyte apoptosis and mesangial proliferation. The renoprotective effects of A manihot are related to inhibition of caspase-3 and caspase-8 overexpression, reduction of the ED1+ and ED3+ macrophages, attenuation of oxidative stress (OS), downregulation of the p38 mitogen-activated protein kinase (p38MAPK) and serine-threonine kinase (Akt) pathways, the suppression of transforming growth factor-β1 (TGF-β1) and tumour necrosis factor-a (TNF-α) protein expression, as well as inhibition the expression of α-smooth muscle actin, phosphorylation-extracellular signal-regulated kinase (p-ERK1/2), nicotinamide adenine dinucleotide phosphate (NADPH) Oxidase 1, NADPH Oxidase 2 and NADPH Oxidase 4[101-104].  In this analysis, the results showed that A manihot added to a RAS blocker could further reduce proteinuria and improve kidney function in DN patients. Four previous meta-analyses[16-19]of A manihot for DN preliminarily reported that A manihot therapy showed great improvements in proteinuria and kidney function, which was consistent with this analysis. The review found that A manihot for DN was well tolerated with minimal ADEs. Since the Huangkui capsule gained national approval from the China Food and Drug Administration in 1999, there have been no reports of severe ADEs. Previous meta-analyses[16-19] of A manihot for DN reported that the most common adverse event was mild to moderate gastrointestinal discomfort, other ADEs such as dizziness, headache, dry mouth were rarely reported. In this analysis, nine types of adverse events were observed, including gastrointestinal discomfort, dry mouth, headache, dizzy, liver injury, hypoglycemia, hyperkalemia, coughing, and hypotension. Well-tolerated gastrointestinal discomfort still was the most common ADE. Other side effects were not frequently reported. Rates of adverse events were not significantly different between study groups except for headache, which was reported to occur more commonly in control group. Although 19 trials of the included trials provided no data for ADEs or ADRs, these studies all clearly reported that A manihot was associated with significant improvements in proteinuria, Scr and clinical symptoms. If confirmed, these results suggested that A manihot might be effective and relatively safe for DN. | 10-13 |
| Limitations | 25 | Although this review is the most comprehensive meta-analysis to date regarding the safety and efficacy of A manihot in addition to a RAS blocker for DN patients, there are limitations that should be considered when interpreting the results.  Firstly, the methodological quality of the included studies was generally low. Most described randomization poorly. None of the trials described allocation concealment. Only one[33]used a placebo control. None of the trials mentioned blinding. One[87] study was given a grade of high risk for attrition bias (incomplete outcome data) due to lack of the information on how missing data was handled in the analysis. This meta-analysis carried a risk of reporting bias because not all studies reported each outcome of interest. All the included trials were single center with generally small sample size, which was likely to make results lack of powder. Heterogeneity was significant among these studies, which weakened confidence in the results. Therefore, the results should be interpreted with caution due to generally low methodological quality and significant heterogeneity.  Secondly, all the identified studies were relatively short, resulting in lack of evidence on the long-term effects of A manihot for DN. In this systematic review, two study[45,91]reported that A manihot was associated with a greater improvement in UAER after 8-week therapy, and the effect could persist for 12 weeks after treatment. However, most of included trials assessed the efficacy immediately after the termination of the treatment period, and did not continue to follow up and investigate the long-term effects that A manihot improved the prognosis of DN. Therefore, long-term studies are required to identify whether the beneficial effects could translate into a reduced risk of clinically meaningful kidney outcomes such as ESRD.  Thirdly, special attention should be paid to ADEs or ADRs. Safety is a fundamental principle in the provision of herbal medicines and herbal products for health care. Current evidence indicated that A manihot combined with a RAS blocker might be relatively safe for DN. 19 of the included trials did not clearly provide data for ADEs or ADRs despite all clear descriptions of great improvements in proteinuria or Scr with A manihot therapy in this review. More detailed study of ADEs and other potential ADEs should be a focus of future studies. | 10-13 |
| Conclusions | 26 | Abelmoschus manihot in addition to a RAS blocker appeared to be effective and safe to further reduce proteinuria and protect kidney function in patients with DN. However, due to the generally low methodological quality, significant heterogeneity and publication bias, high-quality RCTs are required to confirm these findings before the routine use of Abelmoschus manihot can be recommended. | 13 |
| **FUNDING** | | |  |
| Funding | 27 | The study was financially supported by the capital health research and development of special(No. 2016-1-4151); Beijing Natural Science Foundation (No. 7182143); National Natural Science Foundation of China (No. 81774128).. | 13 |

*From:*  Moher D, Liberati A, Tetzlaff J, Altman DG, The PRISMA Group (2009). Preferred Reporting Items for Systematic Reviews and Meta-Analyses: The PRISMA Statement. PLoS Med 6(6): e1000097. doi:10.1371/journal.pmed1000097

For more information, visit: **www.prisma-statement.org**. Page 2 of 2
