# Supplementary material for: Abelmoschus manihot for Diabetic Nephropathy: A Systematic Review and Meta-Analysis
Source: Evid Based Complement Alternat Med. 2019 Apr 18;2019:9679234. doi: 10.1155/2019/9679234 (PMC6500631; doi:10.1155/2019/9679234)
Supplement: Supplementary 2 — File S2: PubMed search strategy. [file 9679234.f2.docx]

Relevant studies will be obtained from English database CENTRAL, PubMed, EMBASE, clinical trials. Chinese Database of Chinese National Knowledge Infrastructure Database (CNKI), Chinese Biomedical Literature Database (CBM), Chinese Scientific Journal Database (VIP), Wan Fang Database.

Search Strategy for PubMed

#1 Abelmoschus[all fields]

#2 Abelmoschus manihot [mh]

#3 Flos Abelmoschus manihot[all fields]

#4 Abelmoschus corolla [all fields]

#5 Abelmoschus moschatus Medicus [mh]

#6 okra [all fields]

#7 ambrette [all fields]

#8 Huangkui [all fields]

#9 Huangkui capsule [all fields]

#10 huangshukui [all fields]

#11 huangshukuihua [all fields]

#12 #1 or #2 or #3 or #4 or #5 or #6 or #7 or #8 or #9 or #10 or #11

#13 diabetic nephropathy [mh]

#14 diabetic nephropath* [all fields]

#15 DN[all fields]

#16 diabetes mellitus [all fields]

#17 diabet* [all fields]

#18 Albuminuria [mh]

#19 kidney disease [mh]

#20 renal disease [all fields]

#21 diabetic kidney disease [mh]

#22 DKD[all fields]

#23 diabetic renal disease [all fields]

#24 #13 or #14 or #15 or #16 or #17 or #18 or #19 or #20 or #21 or #22 or #23

#25 #12 and #24

#26 randomized controlled trial[pt]

#27 controlled clinical trial[pt]

#28 randomized[tiab]

#29 placebo[tiab]

#30 drug therapy[sh]

#31 randomly[tiab]

#32 trial[tiab]

#33 groups[tiab]

#34 #26 or #27 or #28 or #29 or #30 or #31 or #32 or #33

#35 animals[mh] not (humans[mh] and animals[mh])

#36 #34 not #35

#37 #25 and #36
